# The Friendly Policeman

**Published:** 1920-02-14

**Authors:** 


### The Friendly Policeman.

It cannot be doubted that a very great proportion of the young girls in our large cities who "go wrong," do so, not through inherent vice, but simply through ignorance of consequences. Many are friendless and foolish and need nothing more than advice and instruction. It is in this way that the Women Patrols of the Metropolitan Police are doing such invaluable work. Very great care has been taken in selecting these policewomen, for they must have, above all and before all, the extremely rare quality of tact. It is their business to get to know the type of girl to talk with them, to get their confidence, to advise and to help. In the great majority of cases the girls—most of them unhappy and lacking a disinterested friend—are perfectly ready to respond when they are discreetly approached. The patrol finds them safe lodging for the night and takes them to the welfare centre next day, and from there they are found suitable homes. Each case is treated on its merits, and there is no stereotyped procedure. Of course, there are occasionally cases when girls do not respond to this friendly method of handling, and it is then necessary to administer warning and to report the matter at the police station. But the extent of the good work being done can be judged from the fact that within the last six months the Metropolitan Women Patrols have passed on to homes, infirmaries, hostels, refuges, and shelters 250 girls, whose ages varied between 14 and 21.

Of course, this is but a branch of the work done by the Women Patrols, who are trained to deal with any problem affecting the welfare and interests of women and children. Their work is essentially humane and only occasionally punitive, and they have become an extremely valuable institution in the life of London.

